# A trial platform to assess approved SARS-CoV-2 vaccines in immunocompromised patients: first sub-protocol for a pilot trial comparing the mRNA vaccines Comirnaty® and COVID-19 mRNA Vaccine Moderna®

**DOI:** 10.1186/s13063-021-05664-0

**Published:** 2021-10-21

**Authors:** Benjamin Speich, Frédérique Chammartin, Daniel Smith, Marcel P. Stoeckle, Patrizia Amico, Anna L. Eichenberger, Barbara Hasse, Macé M. Schuurmans, Thomas Müller, Michael Tamm, Michael Dickenmann, Irene A. Abela, Alexandra Trkola, Hans H. Hirsch, Oriol Manuel, Matthias Cavassini, Lars G. Hemkens, Matthias Briel, Nicolas J. Mueller, Andri Rauch, Huldrych F. Günthard, Michael T. Koller, Heiner C. Bucher, Katharina Kusejko, in collaboration with the study groups from the Swiss HIV Cohort Study and the Swiss Transplant Cohort Study

**Affiliations:** 1 Department of Clinical Research, Basel Institute for Clinical Epidemiology and Biostatistics, University Hospital Basel, University of Basel, Basel, Switzerland; 2 Nuffield Department of Orthopaedics, Centre for Statistics in Medicine, Rheumatology and Musculoskeletal Sciences, University of Oxford, Oxford, UK; 3 University of Zurich, Institute of Medical Virology, Zurich, Switzerland; 4 Division of Infectious Diseases and Hospital Epidemiology, University Hospital of Basel, University of Basel, Basel, Switzerland; 5 Clinic for Transplantation Immunology and Nephrology, University Hospital Basel, Basel, Switzerland; 6 Department of Infectious Diseases, Inselspital, Bern University Hospital, University of Bern, 3010 Bern, Switzerland; 7 Division of Infectious Diseases and Hospital Epidemiology, University Hospital Zurich, Zurich, Switzerland; 8 Division of Pulmonology, University Hospital Zurich, Zurich, Switzerland; 9 Nephrology Clinic, University Hospital Zurich, Zurich, Switzerland; 10 Clinic of Respiratory Medicine and Pulmonary Cell Research, University Hospital of Basel, Petersgraben 4, 4031 Basel, Switzerland; 11 Clinical Virology, Laboratory Medicine, University Hospital Basel, Basel, Switzerland; 12 Transplantation & Clinical Virology, Department Biomedicine, University Hospital Basel, Basel, Switzerland; 13 Infectious Diseases Service, Lausanne University Hospital and University of Lausanne, Lausanne, Switzerland; 14 Transplantation Center, Lausanne University Hospital and University of Lausanne, Lausanne, Switzerland; 15 Meta-Research Innovation Center Berlin (METRICS-B), Berlin Institute of Health, Berlin, Germany; 16 Meta-Research Innovation Center at Stanford (METRICS), Stanford University, Stanford, CA USA; 17 Department of Health Research Methods, Evidence, and Impact, McMaster University, Hamilton, Canada

**Keywords:** SARS-CoV-2, Randomized controlled trial, HIV, Organ transplant, Platform trial, Vaccine, Protocol

## Abstract

**Background:**

Late 2019, a new highly contagious coronavirus SARS-CoV-2 has emerged in Wuhan, China, causing within 2 months a pandemic with the highest disease burden in elderly and people with pre-existing medical conditions. The pandemic has highlighted that new and more flexible clinical trial approaches, such as trial platforms, are needed to assess the efficacy and safety of interventions in a timely manner. The two existing Swiss cohorts of immunocompromised patients (i.e., Swiss HIV Cohort Study (SHCS) and Swiss Transplant Cohort Study (STCS)) are an ideal foundation to set-up a trial platform in Switzerland leveraging routinely collected data. Within a newly founded trial platform, we plan to assess the efficacy of the first two mRNA SARS-CoV-2 vaccines that reached market authorization in Switzerland in the frame of a pilot randomized controlled trial (RCT) while at the same time assessing the functionality of the trial platform.

**Methods:**

We will conduct a multicenter randomized controlled, open-label, 2-arm sub-study pilot trial of a platform trial nested into two Swiss cohorts. Patients included in the SHCS or the STCS will be eligible for randomization to either receiving the mRNA vaccine Comirnaty® (Pfizer/BioNTech) or the COVID-19 mRNA Vaccine Moderna®. The primary clinical outcome will be change in pan-lg antibody response (pan-Ig anti-S1-RBD; baseline vs. 3 months after first vaccination; binary outcome, considering ≥ 0.8 units/ml as a positive antibody response). The pilot study will also enable us to assess endpoints related to trial conduct feasibility (i.e., duration of RCT set-up; time of patient recruitment; patient consent rate; proportion of missing data). Assuming vaccine reactivity of 90% in both vaccine groups, we power our trial, using a non-inferiority margin such that a 95% two-sided confidence interval excludes a difference in favor of the reference group of more than 10%. A sample size of 380 (190 in each treatment arm) is required for a statistical power of 90% and a type I error of 0.025. The study is funded by the Swiss National Science Foundation (National Research Program NRP 78, “COVID-19”).

**Discussion:**

This study will provide crucial information about the efficacy and safety of the mRNA SARS-CoV-2 vaccines in HIV patients and organ transplant recipients. Furthermore, this project has the potential to pave the way for further platform trials in Switzerland.

**Trial registration:**

ClinicalTrials.govNCT04805125. Registered on March 18, 2021

## Administrative information

Note: the numbers in curly brackets in this protocol refer to SPIRIT checklist item numbers. The order of the items has been modified to group similar items (see http://www.equator-network.org/reporting-guidelines/spirit-2727-statement-defining-standard-protocol-items-for-clinical-trials/)

**Table:** 

Title {1}	A trial platform to assess approved SARS-CoV-2 vaccines in immunocompromised patients: First sub-protocol for a pilot trial comparing the mRNA vaccines Comirnaty® and Covid-19 mRNA Vaccine Moderna® Short title: Immunocompromised Swiss Cohorts Based Trial Platform Acronym: COVERALL (COrona VaccinE tRiAL pLatform)
Trial registration {2a and 2b}.	NCT04805125.
Protocol version {3}	This protocol is based on the master protocol (version 2.1; 07.04.2021) and the first sub-protocol (version 1.1; 07.04.2021) that received final approval on April 19, 2021 from the Ethical committee Nordwest- and Zentralschweiz, Switzerland (2021-000593). The original study protocols are publicly available (https://clinicaltrials.gov/ct2/show/NCT04805125).
Funding {4}	This study receives financial support from the Swiss National Science Foundation (grant # 31CA30_196245). The Swiss HIV Cohort Study (SHCS) and the Swiss Transplant Cohort Study (STCS) are funded by the Swiss National Science Foundation (grant #177499 and #201369) and (grant 33CS30_177522), respectively. Benjamin Speich is supported by an Advanced Postdoc.Mobility grant (P300PB_177933) and a return grant (P4P4PM_194496) from the Swiss National Science Foundation. Roche provides the antibody tests free of charge. The funders have no role in designing, conducting, analysing and publishing trial results.
Author details {5a}	Benjamin Speich^1,2,*^, Frédérique Chammartin^1^, Daniel Smith^3^, Marcel P. Stoeckle^4,^ Patrizia Amico^5^, Anna L Eichenberger^6^, Barbara Hasse^7^, Macé M Schuurmans^8^, Thomas Müller^9^, Michael Tamm^10^, Michael Dickenmann^6^, Irene A Abela^3,7^, Alexandra Trkola^3^, Hans H Hirsch^11^, Oriol Manuel^12,13^, Matthias Cavassini^12^, Lars G. Hemkens^1,14,15^, Matthias Briel^1,16^, Nicolas J Mueller^7^, Andri Rauch^7^, Huldrych F Günthard^3,7^, Michael T Koller^1^, Heiner C Bucher^1^, Katharina Kusejko^3,7^ ^1^ Department of Clinical Research, Basel Institute for Clinical Epidemiology and Biostatistics, University Hospital Basel, University of Basel, Basel, Switzerland ^2^ Nuffield Department of Orthopaedics, Centre for Statistics in Medicine, Rheumatology and Musculoskeletal Sciences, University of Oxford, Oxford, United Kingdom ^3^ University of Zurich, Institute of Medical Virology, Zurich, Switzerland ^4^ Division of Infectious Diseases and Hospital Epidemiology, University Hospital of Basel, University of Basel, Switzerland ^5^ Clinic for Transplantation Immunology and Nephrology, University Hospital Basel, Switzerland ^6^ Department of Infectious Diseases, Inselspital, Bern University Hospital, University of Bern, 3010 Bern, Switzerland ^7^ Division of Infectious Diseases and Hospital Epidemiology, University Hospital Zurich, Zurich, Switzerland ^8^ Division of Pulmonology, University Hospital Zurich, Zurich, Switzerland ^9^ Nephrology clinic, University Hospital Zurich, Zurich, Switzerland. ^10^ Clinic of Respiratory Medicine and Pulmonary Cell Research, University Hospital of Basel, Petersgraben 4, 4031, Basel, Switzerland ^11^ University Hospital Basel, Clinical Virology, Laboratory Medicine, and Transplantation & Clinical Virology, Department Biomedicine, and Infectious Diseases & Hospital Epidemiology, Basel, Switzerland ^12^ Infectious Diseases Service, Lausanne University Hospital and University of Lausanne, Lausanne, Switzerland ^13^ Transplantation Center, Lausanne University Hospital and University of Lausanne, Lausanne, Switzerland. ^14^ Meta-Research Innovation Center Berlin (METRICS-B), Berlin Institute of Health, Berlin, Germany ^15^ Meta-Research Innovation Center at Stanford (METRICS), Stanford University, Stanford, California ^16^ Department of Health Research Methods, Evidence, and Impact, McMaster University, Hamilton, Canada *Corresponding author: Benjamin Speich, PhD, Basel Institute for Clinical Epidemiology and Biostatistics, Department of Clinical Research, University Hospital Basel, University of Basel, Basel, Switzerland, Spitalstrasse 12 4056 Basel Switzerland Email: benjamin.speich@usb.ch
Name and contact information for the trial sponsor {5b}	Sponsor-Investigator: University Hospital Basel Prof. Heiner C. Bucher MD MPH Basel Institute for Clinical Epidemiology & Biostatistics University Hospital Basel Spitalstrasse 12 4056 Basel Switzerland Email: heiner.bucher@usb.ch
Role of sponsor {5c}	The funders have no role in study design; collection, management, analysis, and interpretation of data; writing of the report; and the decision to submit the report for publication.

## Introduction

### Background and rationale {6a}

A new highly contagious coronavirus SARS-CoV-2 has emerged in late 2019 in Wuhan, China, and caused in a 2-month period a pandemic with the highest disease burden in elderly and people with pre-existing medical conditions [1–3]. HIV-positive individuals suffer from multiple comorbidities in particular cardiovascular disease and diabetes due to aging, risk factors for chronic diseases, and lifestyle factors, which put them potentially at a higher risk of complicated coronavirus disease-19 (COVID-19) [4]. Whether poor immune recovery in the presence of antiretroviral therapy is associated with increased risk of SARS-CoV-2 infection and complicated infection is unclear. Likewise, solid organ transplanted patients suffer from multiple chronic conditions like cardiovascular disease and diabetes and are treated with immunosuppressive drugs putting them at risk of multiple infections and severe infection related complications.

In a situation with no known therapy against SARS-CoV-2, clinical research in Switzerland was ill-prepared at the beginning of the pandemic for launching clinical trials for the rapid evaluation of investigative drugs with postulated in vitro activities against SARS-CoV-2. The use of routinely collected data for nesting trials into cohort studies with highly standardized data collection systems and platform trial design allows rapid patient recruitment and efficient and cost-saving data collection for the simultaneous evaluation of several treatment options, features which can expedite trial protocol development and trial implementation in an epidemic situation [5–9].

The advantage of using the existing infrastructure of two well-established cohorts (i.e., Swiss HIV Cohort Study (SHCS) and the Swiss Transplant Cohort Study (STCS)) is that the processes of rigorous follow-up monitoring and collection of high-quality phenotype data is already well established and will be performed entirely within the existing cohorts [10, 11]. The SHCS was established 1988 and is a systematic longitudinal study enrolling HIV-infected patients and is representative for the HIV epidemic in Switzerland [10]. The STCS includes all solid organ transplant recipients in Switzerland after 2008 following the implementation of the Swiss federal act on transplantation of organs, tissues, and cells, March 16, 2007 (Transplantationsverordnung SR 810.211) [11]. The use of highly standardized routinely collected data for conducting intervention trials is a highly efficient and economic approach for trial conduct as exemplified by previous groups [8, 9, 12]. Furthermore, a well-established trial platform would allow us to react quickly in case of any future outbreak, meaning that fewer hurdles need to be overcome to test new interventions.

Since January 12, 2021, two mRNA vaccines against SARS-CoV-2 by Pfizer/BioNTech (Comirnaty®) and COVID-19 mRNA Vaccine Moderna® by Moderna have been licensed in Switzerland and roll-out of vaccines has started [13, 14]. In phase III licensing trials of both vaccines which were both submitted as emergency use authorization (EUA) to the Food and Drug administration (FDA), with parallel submission to the Swiss authorities (i.e., Swissmedic), vaccine efficacy of 95% and 94% were found and both vaccines appeared to be safe. However, estimates of vaccine efficacy were imprecise and with large confidence intervals in the subgroups of individuals most at risk of complications from SARS-CoV-2 infection like the elderly and those with comorbidities. Likewise, data on immune response overall and in subgroups is not yet available [15, 16]. In particular, very few individuals in both trials with HIV infection were included (with no information on the CD4 cell count at the time point of vaccination) and no solid organ recipients were allowed in the trials [15, 16]. On March 22, 2021, Swissmedic approved also the SARS-CoV-2 vaccine from Johnson & Johnson [17]. However, currently (25 August 2021), the two investigational medicinal products (Comirnaty® and COVID-19 mRNA Vaccine Moderna®) are still the only SARS-CoV-2 vaccines that are used in Switzerland [18].

### Objectives {7}

The pilot study will assess the functionality of the trial platform and the comparative effectiveness in terms of antibody response, clinical, and safety outcomes of the first two approved vaccines against SARS-CoV-2 in Switzerland (i.e., Comirnaty® by Pfizer/BioNTech and COVID-19 mRNA Vaccine Moderna® by Moderna). At a later stage, the platform can serve for a larger comparative vaccine trial or can be enriched by new study protocols that investigate new interventions. This can be done, for example in vaccinated individuals in the cohort that did not achieve an immune response after the first vaccine (for more information, see originally approved master and sub-protocol; available here: https://clinicaltrials.gov/ct2/show/NCT04805125).

### Trial design {8}

This is a pilot trial which is based on a trial platform that is integrated into the ongoing routine prospective data collection of two national cohorts, the SHCS and STCS. Eligibility of trial participants will be largely checked from routinely collected cohort data which will also serve as baseline data. Randomization and follow-up data collection of clinical trial endpoint data is done within the trial platform which is linked to the cohort databases. The trial platform is overseen and managed by the data center of the SHCS.

A parallel two-arm (allocation 1:1) open-label randomized controlled exploratory pilot trial comparing the first approved SARS-CoV-2 vaccines is planned to compare the immunogenicity, explore the clinical effectiveness and safety of the first two licensed vaccines against SARS-CoV-2, and assess the functionality of the trial platform. Cohort data centers will identify eligible patients based on routinely collected cohort data, and eligible patients will be contacted for consent for participation prior to randomization. Randomization will be performed through minimization with respect to center, age (< 65, ≥ 65 years old), sex, immune-suppression (< 200; ≥ 200 CD4 cells/μl in SHCS and use of depleting polyclonal antibody preparations for prophylactic induction therapy in addition in addition to standard immunosuppression in STCS patients), and a random element that reduces randomization predictability. The trial platform allows to expand the pilot trial and to add further sub-study protocols to evaluate for example vaccine strategies in patients with first two vaccinations but insufficient or lacking immune response. The COVID-19 pandemic has revealed a need for research infrastructure such as trial platforms, which allow for fast and efficient evaluation of multiple preventive and therapeutic interventions in case of infectious disease outbreaks. Ideally, trial platforms make use of already existing data infrastructures providing routinely collected data (including cohorts and registries). Once implemented, the present protocol is meant to provide orientation and guidance for other trial platforms in Switzerland.

## Methods: participants, interventions, and outcomes

### Study setting {9}

This is a multi-center study recruiting patients from 4 of the 7 study centers of the SHCS (i.e., University Hospital Basel, University Hospital Zurich, University Hospital Bern, and CHUV Lausanne) and 3 of the 6 study centers of the STCS (i.e., University Hospital Basel, University Hospital Zurich, and CHUV Lausanne). Both cohorts are representative for individuals living with HIV and solid organ transplants, respectively.

### Eligibility criteria {10}

#### Inclusion criteria

- All patients with either a chronic HIV infection or recipients of solid organs registered with informed consent from the SHCS or STCS cohorts
- ≥ 18 years
- Patients with solid organ transplantation of lungs or kidneys at least one month post-transplantation with a prednisone dose of 20 mg or less
- Additional consent for participation in the pilot trial
- COVID-19 vaccination recommended by treating physician

#### Exclusion criteria

- Pregnancy
- Acute symptomatic SARS-CoV-2 infection, influenza, or other acute respiratory tract infection
- Known allergy or contra-indications for vaccines or any vaccine components
- Any emergency condition requiring immediate hospitalization for any condition
- Patients with previous polymerase chain reaction (PCR) documented SARS-CoV-2 infection and/or documented antibodies less than 3 months prior to randomization
- Patients with solid organ transplantation (lung or kidney) with the following conditions:
- Solid organ transplant recipients less than one month post-transplantation
- Solid organ transplant recipients with the use of T cell depleting agents in the last 3 months (i.e., induction treatment in standard risk or high-risk immunological situation or rejection treatment)
- Solid organ transplant recipients with the need of pulse corticosteroids (> 100 mg prednisone or equivalent) in the last 1 month or rituximab in the last 6 months
- Solid organ transplant recipients with the need of any kind of chemotherapy treatment

### Who will take informed consent? {26a}

Cohort participants will be contacted by the local centers (i.e., be their treating cohort physicians or by delegated staff) and invited to get an appointment at the site vaccination center for a vaccination against SARS-CoV-2 or get information about vaccines and the trial during cohort visits. When a potentially eligible patient is visiting a local cohort center, the investigators will explain the nature of the study, its purpose, the procedures involved, the expected duration, the potential risks and benefits, and any discomfort it may entail (this step can also be conducted through a phone call if preferred). Each participant will be informed that the participation in the study is voluntary and that he/she may withdraw from the study at any time and that withdrawal of consent will not affect his/her subsequent medical assistance and treatment. Patients will have sufficient time to ask questions and to consent to trial participation (they are allowed to consider trial participation at a sub-sequent visit).

### Additional consent provisions for collection and use of participant data and biological specimens {26b}

Participants have already provided written informed consent that their data, stored plasma, and blood cell samples that are routinely collected within the Swiss HIV Cohort Study and the Swiss Transplant Cohort Study are used by authorized researchers of both cohorts other than their treating physician in the frame of the ongoing cohort studies.

## Interventions

### Explanation for the choice of comparators {6b}

BNT162b2 (30 μg) commercial name Comirnaty® developed by Pfizer/BioNTech was the first approved SARS-CoV-2 vaccine in Switzerland (approved on 19 December 2020) [19]. It is administered intramuscularly (IM) as a series of two 30 μg doses of the diluted vaccine solution (0.3 mL each) according to the following schedule: a single dose followed by a second dose 28 days later [20]. The Comirnaty® vaccine was the first approved SARS-CoV-2 vaccine in Switzerland. The storage and handling of the vaccine is quite challenging (i.e., Comirnaty® is supplied as a multiple-dose (5-dose) vial containing a frozen (between − 80 °C and − 60 °C suspension that is preservative-free.) BNT162b2 must be thawed and diluted in its original vial with 1.8 mL of sterile 0.9% sodium chloride injection, prior to administration. After dilution, the vial contains 5 doses of 0.3 mL per dose (Swiss guidelines allow for preparation of 6 doses from one vial) and must be stored between 2 °C and 25 °C and used within 6 h from the time of dilution [21]).

### Intervention description {11a}

The COVID-19 mRNA Vaccine Moderna® was the second approved SARS-CoV-2 vaccine in Switzerland (approved 12 January 2021) [22, 23]. The producer recommends to apply two doses, administering the second dose 28 days after the first dose [24]. The handling and storage of the Moderna vaccine is somewhat easier compared to the Comirnaty® vaccine (i.e., storage required at − 25 °C to − 15 °C; can be stored at 2 °C to 8 °C, protected from light, for up to 30 days and is supplied as a multi-dose vial (10 doses, Swiss guidelines allow for the preparation of 11 doses from one vial) [25]. To compare the effectiveness in terms of serological immune response of approved SARS-CoV-2 vaccines in immunocompromised patients, we aim to demonstrate that the second vaccine available in the market is no worse than the comparator by more than 10%.

### Criteria for discontinuing or modifying allocated interventions {11b}

Trial participants are vaccinated according to the producers’ dosing recommendations. No adaptation of vaccine dosing is planned. In case of a serious adverse event following the application of a first dose, the second dose will not be given. Enrolled patients have always the opportunity to withdraw from the trial without mentioning any specific reason at any time. In addition, patients can also state that they do not wish to be contacted for the study anymore and also that their physician shall not contact them for outcome assessment.

### Strategies to improve adherence to interventions {11c}

As the vaccines are given by health personnel, the adherence to the intervention will be assessed and documented as required by the vaccine producers and the Swiss Federal Office of Public Health. In case that patients miss their appointment for the second vaccine, their cohort physician will contact them by phone and/or email to make sure that they receive the second vaccine dose or to assess the reason why they wish to withdraw.

### Relevant concomitant care permitted or prohibited during the trial {11d}

Concomitant drugs for patients included into the trial are recorded within the routine cohort based data collection structure. Physicians are free in the choice of any concomitant drugs for the treatment of HIV, immunosuppressive drugs for transplanted patients, or any other drugs used for the treatment of additional chronic conditions and take decisions for their use according to best clinical judgment and treatment guidelines. There are no particular drugs that are not permitted for their use during vaccination and follow-up. The use of any drugs for the treatment of local or systemic adverse effects of vaccines is according to clinical judgment by clinicians.

### Provisions for post-trial care {30}

Patients will continue to be routinely followed by biannual visits in the SHCS and annual visits in the STCS and are cared at cohort centers or delegated cantonal hospitals and private physicians that provide specialized patientcare, conduct cohort visits and collect data.

### Outcomes {12}

#### Immunological outcomes

The primary immunological endpoint are antibodies (yes/no) to the SARS-CoV-2 spike (S1) protein receptor binding domain in human serum and plasma assessed 12 weeks after the first vaccination by the commercial immunoassay Elecsys® Anti-SARS-CoV-2 S for the in vitro quantitative determination [26]. This assay detects pan-Ig antibody response (pan-Ig anti–S1-RBD) and allows for a quantitative assessment of the serological response of the participants at baseline and 3 months after vaccination (primary outcome). An antibody response will be considered as positive using the threshold ≥ 0.8 units/ml, as defined by the manufacturer. Additionally, we will qualitatively measure anti-Nucleocapsid (N) responses at baseline with the Elecsys® Anti-SARS-CoV-2 N assay to gain a broader insight on the immune response of the participants.

Furthermore, SARS-CoV-2-binding antibody responses of the participants will be assessed by analyzing IgM, IgA, and IgG responses to a wider range of SARS-CoV-2 antigens (RBD, S1, S2, and N) with a bead-based multiplex immunoassay using the Luminex technology© termed Antibody CORonavirus Assay (ABCORA) 2.0. This assay was established at the Institute of Medical Virology (IMV), University of Zurich. The ABCORA 2.0 test has an advantage over commercial available tests, as it allows a parallel assessment of IgG, IgM, and IgA reactivity to multiple antigens enabling a high-dimensional temporal resolution of antibody responses and a differentiation between humoral responses to an infection and vaccination [27].

#### Clinical outcomes

The clinical effectiveness endpoints are (according to FDA recommendations for phase III COVID-19 vaccine licensing trials (28)) the following:

- Newly PCR-confirmed asymptomatic SARS-CoV-2 infections (identified by the presence of anti–SARS-CoV-2 nucleocapsid antibodies or SARS-Cov-2 PCR or rapid antigen test) and no related symptoms (i.e., fever or chills, cough, shortness of breath or difficulty breathing, fatigue, muscle or body aches, headache, new loss of taste or smell, sore throat, congestion or runny nose, nausea or vomiting, and diarrhea) at any time point within 48 weeks following randomization.
- Newly PCR-confirmed symptomatic SARS-CoV-2 infections with at least one of the following symptoms (i.e., fever or chills, cough, shortness of breath or difficulty breathing, fatigue, muscle or body aches, headache, new loss of taste or smell, sore throat, congestion or runny nose, nausea or vomiting, and diarrhea) at any time point within 48 weeks following randomization.
- Severe SARS-CoV-2 infections with respiratory failure, evidence of shock (as diagnosed by a treating physician), clinically significant acute renal, hepatic, or neurologic dysfunction; admission to an intensive care unit; and death at any time point within 48 weeks following randomization.
- COVID-19 burden of diseases (BOD), a composite of the above endpoints. The BOD will be scored as by using 0 for no SARS-CoV-2, 1 for non-severe SARS-CoV-2, and 2 for severe SARS-CoV-2 [28].

An additional clinical effectiveness endpoint is patient-reported asymptomatic or symptomatic infections of household members.

All clinical outcomes will be measured in the trial database during a follow-up period of 48 weeks after randomization and vaccination. Patients will be further followed in the frame of the already existing cohort studies (i.e., STCS and SHCS).

#### Safety outcomes

Collection of solicited local and systemic adverse events will for feasibility reasons be reduced to:

- Any local symptom (redness or swelling or prolonged pain at injection site) limiting continuation of normal daily activities during the first 7 days after vaccination
- Any systemic symptom (fever, generalized muscle or joint pain) limiting continuation of normal daily activities during the first 7 days after vaccination
- Any vaccine related symptom leading to contacting a physician during the first 7 days after vaccination

#### Feasibility outcomes for the trial platform

• Duration of RCT set up (i.e., time from deciding which interventions will be tested until the first patient is randomized)

• Time of patient recruitment (i.e., from activation of first study site until (i) 40 patients and (ii) 380 patients are randomized)

• Patient consent rate (i.e., proportion of patients giving informed consent out of approached eligible patients)

• Proportion of missing data for all baseline variables from routinely collected cohort data

• Proportion of missing data for all clinical outcomes from routinely collected cohort data and outcome data that is collected in the trial platform (clinical outcomes are listed above)

### Participant timeline {13}

The participant timeline is presented in the study schedule (Table 1).

**Table 1:** Study schedule

		Study period				
	Eligibility	Enrollment, randomization and vaccination	Post-randomization			
Time point	Approximately 12 weeks	Day 0	Week 4	Week 12 (± 7 days)	Week 48 (± 7 days)	Continuous follow-up in the frame of the cohort
Enrollment:						
Eligibility screen^a^	X^a^					
Eligibility assessment	X					
Informed consent		X^a^				
Allocation		X^b^				
Interventions:						
Intervention COVID-19 mRNA Vaccine Moderna®		X	X			
Control Comirnaty® (Pfizer/BioNTech vaccine)		X	X			
Assessments:						
Baseline variables (will be exported from routine cohort data)Baseline blood sample	X					
IgG neutralizing antibodies post randomization but prior to vaccination		X				
Clinical outcomes:	X^e^	X^e^				
- Immune response IgG neutralizing antibodies		X		x^c^		
- Newly confirmed asymptomatic SARS-CoV-2 infection				x^c^	x^c,d^	x^d^
-Newly confirmed symptomatic SARS-CoV-2 infection				x^c^	x^c,d^	x^d^
- Severe COVID-19 infection with respiratory failure, evidence of shock, organ failure, admission to ICU, or death				x^c^	x^c,d^	x^d^
- Adverse events from vaccines				x^c^		
-Serious adverse events			x^c,d^	x^c^	x^c,d^	x^d^
- PCR confirmed symptomatic infections of household members				x^c^	x^c.d^	x^d^

^a^Continuous assessment and selection from routinely collected cohort data

^b^Enrollment and concealed allocation of patients with informed consent will be performed during the same visit when the study is explained to the patient or, if preferred, during a separate arranged study visit. Not all study centers will have both vaccines available at each vaccination day. This probably requires us to randomize some patients as soon as they give consent to a specific vaccination day on which their allocated vaccination will be applied some days before applying the vaccine

^c^Assessed in separate data entry form for the trial

^d^Assessed by routine data collection from cohort data base

^e^Baseline blood samples will be taken from previous cohort visits (no longer than 6 months) or during cohort and randomization visits at day 0

#### Eligibility (and eventual enrollment randomization)

Visit 1; day −90 to 0: All patients from the two cohorts (i.e., SHCS and STCS) will prospectively be assessed for eligibility based on the cohort study based trial platform. Eligible patients will be either contacted ahead by cohort centers and informed about the trial and availability of vaccines or directly informed during in between visits or cohort study visits. Based on the patients preferences, they will have the option to start participating in the study (i.e., sign consent form, be randomized and receive allocated treatment intervention) during the same visit or at a separate outpatient visit (see also “Study schedule”).

#### Enrollment and randomization

Visit 2; day 0: The patient will return the signed consent form which will also be signed by the treating physician. The patient will then be randomized by the treating physician and receive the study medication (applied by treating physician or delegated personnel). In addition, they will provide a blood sample for the baseline of the immunological outcomes. For patients who were already enrolled and randomized during visit 1, visit 2 will be skipped (see also “Study schedule”). For women in childbearing age, it is required to make sure that they are not pregnant (i.e., conduct a pregnancy test) before they get vaccinated and a contraception is required for 12 weeks after the first vaccination dose.

#### Post randomization, week 4

Patients will receive the second vaccination dose.

#### Post randomization, week 12

Patients will be asked 3 questions relating to potential effects or side effects of the vaccination during the first week following each vaccination dose. Occurrence of asymptomatic or symptomatic SARS-CoV-2 infection, serious SARS-CoV-2 infection, and hospitalization for any reason will be investigated; the dates of events will be requested and entered into the trial data collection form. A blood sample will be taken to assess the immunological outcomes. All routine data and blood samples following protocols of the SHCS and STCS will be collected as usual and entered into the respective cohort database. A blood sample will be taken for investigating post-vaccination immune status at week 12 in all patients.

#### Post randomization, week 48

Occurrence of asymptomatic or symptomatic SARS-CoV-2 infection, serious SARS-CoV-2 infection, and hospitalization for any reason will be investigated; the dates of events will be requested and entered into the trial data collection form. All routine data and blood samples following protocols of the SHCS and STCS will be collected as usual and electronically forwarded to the respective cohort databases.

### Sample size {14}

Phase 1 to phase 3 trials of COVID-19 mRNA Vaccine Moderna® and Comirnaty® (Pfizer/BioNTech) report immunological response titers that are not comparable due to the different assays that were used. Moderna uses an in-house developed ELISA assay, while Pfizer/BioNTech uses an in-house developed Luminex assay. Currently available results show that titers are high at 4 weeks post vaccination and the proportion of patients that is reactive to vaccination is close to 100% [29, 30]. However, no data is available for an immunocompromised population such as this study population. By assuming vaccine reactivity of 90% in both vaccine groups, we power our non-inferiority trial so that a 95% two-sided confidence interval excludes a difference in favor of the reference group of more than 10%. A sample size of 380 (190 in each treatment arm) is required for a statistical power of 90% and a type I error of 0.025. Sample size was calculated using the “ssc_propcomp” function of the R statistical software package “SampleSize4ClinicalTrials” [31].

### Recruitment {15}

Specific eligibility criteria can be defined and implemented by the trial platform data base which is based on all clinical and laboratory cohort data and managed by the cohort data centers. Both cohorts have developed a clinical information system that summarizes all relevant clinical and laboratory information for patients and widely serves as a clinical management and decision support system. This system allows to flag all eligible patients by center and center physician and to make this information available for the pilot trial in the clinical information and data entry system. Thus, trial eligibility information will appear on the online cohort data entry form which is used by all participating cohort centers and physicians for data entry during cohort visits. When an eligible patient is coming to a planned cohort visit or in between consultations the treating physician will recognize the patient as eligible when opening the patient specific data entry form and initiate the recruitment process. In case of logistic needs, patients may also be actively contacted, informed, and invited to participate in the trial. This is in line with the priority list established by the Swiss Federal Office of Public Health for vaccination of high risk patients for COVID-19 infection [22]. The eligibility checks, consent process, and randomization will be embedded into existing regular cohort or scheduled in between visits and done in a separated Good Clinical Practice conform clinical trials data collection system that is linked to both cohorts. The treating physician will explain the currently ongoing study to eligible patients and inform them about potential risks and benefits. Both cohorts (i.e., SHCS and STCS) have together approximately 15,000 patients under active follow-up. It will be key that the trial is started before the majority of these patients have received a SARS-CoV-2 vaccine outside of this trial. We believe that a target sample of 380 patients participants is feasible, which represents 32 patients distributed across 7 participating centers

## Assignment of interventions: allocation

### Sequence generation {16a}

For randomization, treating physicians will use a link function in the clinical information and data entry form and be linked to the web-based platform trial data entry and randomization page. After rechecking all inclusion and exclusion criteria and after having the trial participation consent formed signed, patients will be assigned to study groups using minimization with a random element across a number of stratification factors to control for imbalance in each treatment arm (stratification factors study center, age group, sex and presence of comorbidities). Randomization will be done separately for the two cohorts (STCS, SHCS). In case a patient is included in both cohorts, he/she will be randomized within the cohort that the treating physician deems as most relevant for the current immunodeficiency. Patients not consenting to participate will be vaccinated according to clinicians’ judgment and be followed within the cohorts. The trial platform will allow for adaptive randomization for future trials.

### Concealment mechanism {16b}

The randomization will be implemented into Research Electronic Data Capture (REDCap). The REDCap platform offers an audit trail to maintain a record of initial entries.

### Implementation {16c}

The allocation sequence will be implemented by the data management team of the two study cohorts and the trial statistician. Patients will be contacted by study physician and local investigators (or designated personnel) will assign patients to interventions.

## Assignment of interventions: blinding

### Who will be blinded {17a}

As we plan to embed the pilot trial as good as possible into the clinical routine and within the cohort study infrastructure, we do not plan to blind treating physicians or patients. Further, also, outcome assessors will not be actively blinded. Laboratory personnel conducting immunological tests (primary outcome) will be blinded. Large part of the outcomes will be collected through routinely collected data. Analysis of immunological parameters will be blinded in regard to treatment groups. All the critical clinical endpoints of severe COVID-19, deaths, and serious adverse events will be adjudicated by an infectious disease physician blinded to vaccine allocation.

### Procedure for unblinding if needed {17b}

Not applicable as the study has an open design.

## Data collection and management

### Plans for assessment and collection of outcomes {18a}

#### Assessment of outcomes

##### Assessment of immunological outcomes

We will assess pan-IgG anti S (RBD) (quantitative) and anti-N antibodies (qualitative) with the commercial Elecsys® Anti-SARS-CoV-2 tests at baseline and at 12 weeks following randomization and vaccination (primary immunological endpoint).

We will assess SARS-CoV-2-binding antibody responses of the participants by analyzing the IgM, IgA, and IgG responses to a wider range of SARS-CoV-2 proteins (S1, S2, RBD and N using a newly developed assay Antibody CORonavirus Assay (ABCORA) 2.0 [27] established at the Institute of Medical Virology (IMV), University of Zurich (secondary immunological endpoint). The ABCORA 2.0 test allows for a parallel assessment of IgG, IgM, and IgA reactivity to multiple antigens enabling a high-dimensional temporal resolution of antibody responses and a dissection between humoral responses to an infection and vaccination. ABCORA2.0 antibody test has proven to be more sensitive than the Elecsys® Anti-SARS-CoV-2 S antibody test [27].

Patients will be invited to see their treating physician 12 weeks after randomization (see participant timeline). During baseline and follow-up visits, a blood sample will be taken (EDTA (ethylenediaminetetraacetic acid) blood (2 × 7.5 ml)) and assessed for the above listed antibodies using the following methods: (i) 1 ml for Elecsys® Anti-SARS-CoV-2 N and S tests (performed locally at each laboratory) and (ii) 2 ml EDTA Plasma for ABCORA 2.0 test (performed at IMV, University of Zurich).

EDTA blood should be processed within 24 h, and EDTA plasma should be stored at − 20 °C. For ABCORA 2.0 antibody measurements, plasma samples will be collected at each center and a collective shipment will be sent after baseline and at 12 weeks after randomization and vaccination to the IMV, University of Zurich. One sample will be stored according to SHCS and STCS protocols for later possible analyses at the centers of University Hospital Zurich.

##### Assessment of clinical endpoints

Patients are informed to contact center physicians in case an asymptomatic or symptomatic SARS-Cov-2 infections was diagnosed outside the settings of participating cohort centers. Clinical endpoints will be assessed at 12 and 48 weeks, and during each cohort visits, that fall in between the trial specific visit dates.

- Newly PCR-confirmed asymptomatic SARS-CoV-2 infection (identified by the presence of anti–SARS-CoV-2 nucleocapsid antibodies, or PCR or rapid antigen test) and no related symptoms (i.e., fever or chills, cough, shortness of breath or difficulty breathing, fatigue, muscle or body aches, headache, new loss of taste or smell, sore throat, congestion or runny nose nausea or vomiting, and diarrhea) at any time during the 48 weeks of follow-up.
- Newly PCR-confirmed symptomatic SARS-CoV-2 infection with at least one of the following symptoms (i.e., fever or chills, cough, shortness of breath or difficulty breathing, fatigue, muscle or body aches, headache, new loss of taste or smell, sore throat, congestion or runny nose nausea or vomiting, and diarrhea) at any time during the 48 weeks of follow-up
- Severe SARS-CoV-2 infection with respiratory failure, evidence of shock (as diagnosed by a treating physician), clinically significant acute renal, hepatic, or neurologic dysfunction; admission to an intensive care unit; or death at any time during the 48 weeks of follow-up.
- COVID-19 burden of diseases (BOD), a composite of the above endpoints. The BID is will be scored as by using 0 for no SARS-CoV-2, 1 for non-severe SARS-CoV-2, and 2 for severe SARS-CoV-2.

During all visits at the trial centers (study specific at 12 and 48 weeks, as well as cohort visits), a PCR SARS-COV-2 test will only be conducted if the treating physician would also conduct such a test during clinical routine (e.g., exposure to infected individual or suggestive or unclear symptoms). PCR tests done in between independently will be recorded with dates and results. In case of a positive test result, physicians will check for symptoms and patients will be followed-up according to clinical routine and physicians’ judgment.

The burden of diseases endpoint will be assessed by combining the before listed outcomes (i.e., no CSARS-CoV-2-infection, non-severe SARS-CoV-2 infection).

- Patient-reported asymptomatic or symptomatic infections of household members.

Patients will be asked at the 12-week and 48-week visit if a household member was diagnosed with COVID-19.

##### Assessment of safety outcomes

All safety outcomes will be assessed during the 12-week visit. Patients will be asked if they had any local symptoms which limited their daily activities during the first 7 days after each vaccination dose.

### Plans to promote participant retention and complete follow-up {18b}

In case that patients miss their appointment for the second vaccine, their cohort physician will contact them by phone and/or email to make sure that they receive the second vaccine dose or to assess the reason why they wish to withdraw. Each participant will be informed that the participation in the study is voluntary and that he/she may withdraw from the study at any time and that withdrawal of consent will not affect his/her subsequent medical assistance and treatment. If a patient withdraws from the trial or is lost to follow-up, the reason for drop-out will be reported in the corresponding electronic case report form (eCRF). If the patient withdraws from the cohort, a cohort specific stop follow-up form is in place.

### Data management {19}

Study data will be captured via REDCap, based at the data center of the SHCS. The data collected is entered into the study eCRF. Baseline data available in the two participating cohorts (SHCS and STCS) is transferred directly from the cohorts into REDCap. The REDCap platform offers an audit trail to maintain a record of initial entries and any changes made, time and date of entry, and username of the person authorizing entry or change. The principal investigator at the study site will be responsible for assuring that the data entered into the eCRF is complete and accurate and that the entry and updates are performed in timely manner.

### Confidentiality {27}

Direct access to source documents will be permitted for purposes of monitoring, audits, and inspections. Study data entered into the eCRF is only accessible by authorized persons. Once all data is entered into the REDCap platform and monitoring is completed, the database will be locked and closed for further data entry. The complete dataset is then exported and transferred to the study statistician as well as the principal investigator through a secure channel.

### Plans for collection, laboratory evaluation, and storage of biological specimens for genetic or molecular analysis in this trial/future use {33}

Samples for the ABCORA2.0 and Elecsys® Anti-SARS-CoV-2 S and N tests will be locally stored at the University Hospital virology laboratories and will be shipped by two shipments for all samples collected at baseline after completion of recruitment and after completion of the 3 months follow-up in all patient for all samples collected at 3-month follow-up. Samples will be sent to the Institute of Medical Virology, University of Zurich, and will be evaluated centrally. Samples used for diagnostic purposes will be stored for 5 years.

## Statistical methods

### Statistical methods for primary and secondary outcomes {20a}

Patients will be analyzed according to their allocated randomization group (intention to treat {ITT}). The pilot trial will inform us on the amount of missing variables. Immunological outcomes at baseline and 12-week follow-up will be reported as frequency and percentage of positive serologic immune response for both vaccine arms. The primary objective is to assess the non-inferiority of the second licensed vaccine versus the first licensed vaccine in regards to the presence of pan-IgG antibody, as measured by the Elecsys® Anti-SARS-CoV-2 S immunoassay. Difference in primary outcome among the two vaccine arms will be assessed by a two-sided 95% confidence interval, showing a credible range for the true difference between the second licensed vaccine and the first licensed vaccine. Non-inferiority will be established at the 2.5% significance level, if the lower limit of a 95% two-sided Wald confidence interval for the difference (second licensed–first licensed) is above -10%, where 10% is the pre-defined non-inferiority margin. In a sensitivity analysis, the primary outcome will be assessed, excluding patients that had already of pan-IgG antibody at baseline (threshold ≥ 0.8 units/ml).

A secondary analysis will assess the non-inferiority of the second licensed vaccine versus the first licensed vaccine in regard to the presence of IgM, IgA, and IgG as measured by the ABCORA 2.0 assay.

Clinical outcomes related to COVID-19 infection at 48 weeks and patient-reported COVID-19 infection of household members will be reported as frequency with percentage for the different treatment arms.

### Interim analyses {21b}

No interim analysis will be done.

### Methods for additional analyses (e.g., subgroup analyses) {20b}

We will report frequency and percentage of positive serological immune response for both vaccine arms among different pre-specified subgroups of patients most prone to complicated infections with SARS-CoV-2.These are for HIV-positive individuals, patients with less than 200 CD4cell/μL, unsuppressed HI viral load, male gender, age group 60 to 69, 70 older, and history of cardiovascular diseases or present metabolic syndrome, and for transplanted individuals, intense (triple or quadruple immunosuppressive regimen) versus less intense immunosuppressive therapy (dual immunosuppressive regimen), male gender, age group 60 to 69, 70 older, and history of cardiovascular diseases or presence of metabolic syndrome. Furthermore, we will analyze the data separately for the cohort studies and the transplanted organs.

### Methods in analysis to handle protocol non-adherence and any statistical methods to handle missing data {20c}

We will report the number of drop-outs and reasons for drop-outs for each treatment arm separately. Reasons for drop-outs will be carefully explored for a deeper understanding of the drop-outs. In this pilot study, patients with missing outcomes will be dropped from the clinical outcome assessments. Patient characteristics of the dropped population will be compared to patient characteristics of the trial participants that have an outcome to avoid further bias.

### Plans to give access to the full protocol, participant level-data, and statistical code {31c}

The study protocols (master and sub-protocol) as approved by the ethical committee are publicly available (https://clinicaltrials.gov/ct2/show/NCT04805125). Since data is collected in the frame of the cohort studies (SHCS and STCS), the data sharing rules of these cohorts apply. Access to cohort data can be provided after a respective proposal is submitted to the corresponding scientific boards (see full protocols for more information; https://clinicaltrials.gov/ct2/show/NCT04805125). The statistical code will be made available.

## Oversight and monitoring

### Composition of the coordinating center and trial steering committee {5d}

The core group of this trial will have a weekly meeting to discuss day-to-day questions regarding the planning and conduct of the trial. In addition, a weekly meeting with the data management team is held, to resolve open questions. The principle investigator and the trial manager are in regular exchange with the local investigators to resolve queries and questions.

Data monitoring will be exclusively done centrally within the infrastructure and processes established by each cohort. Weekly check-up of data entries into the clinical trial platform is done by the central data manager and the trial biostatistician. The following central data monitoring steps are planned.

Patient recruitment will be centrally controlled and compared with the total number of eligible patients per center and cohort and the number of patients enrolled.

#### Selection bias

Baseline characteristics of patients included into the trial and those vaccinated outside the trial can be compared to check whether the trial population is representative in respect to the cohort population.

#### Trial inclusion

The responsible biostatistician/data manager will regularly check whether no patients violating an inclusion/exclusion criteria is recruited into the trial.

#### Population description

The responsible biostatistician/data manager will check whether according to the baseline cohort data used for the pilot trial is complete and no data is missing (see also feasibility endpoints).

#### Intervention

For each patient included into the trial, local data managers and the central data managers will check whether patients received the same first and booster vaccines within the 4 week period. Co-medication will be recorded within the cohort data base infrastructure.

#### Primary endpoint

Site responsible investigators will regularly check that all samples are taken and stored at the site laboratories and shipped to the central laboratory. The trial data manager/biostatistician will control that all samples results are adequately entered into the trial platform data base.

#### Secondary endpoints

Local and central cohort data managers will regularly check that all secondary endpoints are completely entered into the trial platform and into cohort data base.

On-site monitoring visits are possible according to Good Clinical Practice, in case larger quality breaches are identified during the central data monitoring.

### Composition of the data monitoring committee, its role and reporting structure {21a}

An independent data safety monitoring board (IDSMB) has been established (see original master protocol; available here: https://clinicaltrials.gov/ct2/show/NCT04805125) which will be advisory to the Trial Committee that constitutes of the PI and the co-investigators. The IDSMB will be regularly informed about any safety aspects and on serious adverse events from vaccinations from the first sub-study protocol, and it will provide the primary investigator and the co-investigators with recommendations about the further conduct of the trial.

### Adverse event reporting and harms {22}

For reporting of serious adverse events, we will adhere to the ICH E2A guidelines [32] and define a serious adverse event (experience) or reaction “as any untoward medical occurrence that at any dose results in death, is life-threatening, requires inpatient hospitalization or prolongation of existing hospitalization, results in persistent or significant disability/incapacity, or is a congenital anomaly/birth defect.” Exemptions will be SARS-CoV-2-related deaths or SARS-CoV-2-related hospitalizations, which are among the assessed clinical outcomes. The term “life-threatening” in the definition of “serious” refers to an event in which the patient was at risk of death at the time of the event; it does not refer to an event which hypothetically might have caused death if it were more severe. Any serious adverse events and suspected unexpected serious adverse events must be reported within 7 days to the sponsor-investigator. The sponsor-investigator will assess the adverse event and report to all local investigators as well as to the corresponding ethical committee (for more information, see originally approved master and sub-protocol; available here: https://clinicaltrials.gov/ct2/show/NCT04805125).

### Frequency and plans for auditing trial conduct {23}

The study documentation and the source data/documents are accessible to auditors/inspectors and questions are answered during inspections. All involved parties must keep the patient data strictly confidential. Both cohorts have an annual local inspection at each center in place with random charts checks for consistency of all data entries.

### Plans for communicating important protocol amendments to relevant parties (e.g., trial participants, ethical committees) {25}

Substantial amendments are only implemented after being discussed among the study investigators and after approval of the Competent Ethics Committee (CEC). The start or closure of a sub-study will be added to the master-protocol as an amendment and are reported to the CEC. Under emergency circumstances, deviations from the protocol to protect the rights, safety, and well-being of human subjects may proceed without prior approval of the sponsor and the CEC. Such deviations shall be documented and reported to the sponsor and the CEC as soon as possible. All minor amendments that have no direct effect on the study conduct and are of primarily administrative nature will be recorded. These changes will be reported to the CEC on the Principal Investigator discretion. All changes will be documented in the final results publication of the pilot study.

### Dissemination plans {31a}

All trials that are set-up within the trial platform and results from sub-studies will be published (also negative results or if a trial has to be discontinued) open access in peer-reviewed journal publications. The original approved trial protocols (master protocol and subsequent sub-protocols) will be made publicly available on clinical trial registration sites or on Open Science Framework. Authorship to publications will be granted according to the rules of the International Committee of Medical Journal Editors (ICMJE).

## Discussion

The set-up of a well-planned RCT is time intensive and requires large resources that are scarce in the non-industry setting [33–36]. Smarter ways to conduct RCTs should be available so that we are able to answer the most urgent clinical questions within RCTs. Platform trials have an efficient study design and can solve some of the problems connected to traditional RCTs. In contrast to commonly used RCTs, where a separate infrastructure is set up for each RCT, trial platforms allow to address multiple research questions in one administrative trial structure [6]. For example, the RECOVERY (Randomized Evaluation of COVID-19 Therapy) platform has constantly added and assessed new promising interventions to treat COVID-19 and has published results on three interventions (i.e., dexamethasone, hydroxychloroquine, and ritonavir boosted lopinavir) within less than 12 months after the SARS-CoV-2 pandemic outbreak [37]. With the COVERALL study, we plan to implement a platform trial which takes advantage of the excellent data infrastructure of two cohorts of immune compromised hosts for investigating vaccine efficacy against SARS-COV-2 in very vulnerable patient populations in the Swiss setting (i.e., assessing all the processes needed to implement a platform study, e.g., how to receive ethical approval) and to evaluate the functionality of the platform trial within a pilot study. We believe that the trial platform will have the following advantages: (i) identifying potential participants through effective pre-screening; (ii) provide an efficient data entry system which takes advantage of already available data (baseline data available in the two participating cohorts (SHCS and STCS) will be transferred directly from the cohorts into REDCap); and (iii) follow-up, sampling, and biobanking will be facilitated by existing infrastructure.

The planned study will compare the efficacy of the two first in Switzerland approved SARS-CoV-2 vaccines in immunosuppressed patients (i.e., patients from the SHCS and the STCS). A practical issue that we will face is that not all study centers will have both vaccines available on each vaccination day. This probably requires us to randomize some patients as soon as they give consent to a specific vaccination day on which their allocated vaccination will be applied. In theory, this could cause that some patients will miss their intervention. However, due to the close contact to the patients through the cohorts, we know that patients are highly motivated to receive the vaccines and we are confident that the proportion of missed first doses will be minimal.

A further limitation of this study is that a recently published research letter (i.e., after ethical approval of this study) revealed a low immune response was found in solid organ transplant patients after receiving a mRNA SARS-CoV-2 vaccine [38]. The results indicate that the assumptions in our sample size calculation (i.e., assuming vaccine reactivity of 90% in both vaccine groups) were probably too optimistic. Nevertheless, we are convinced that providing additional data in this vulnerable population will be crucial to assess if further actions (e.g., a third vaccine booster) are required.

By assessing immunological response with the multi-parameter SARS-CoV-2 seroprofiling test, termed ABCORA 2.0, we will be able to simultaneously record antibody responses to a range of SARS-CoV-2 antigens and different Ig classes in a single assessment. This highly accurate test not only yields a detailed picture on the SARS-CoV-2 serology status but also allows for distinguishing between antibody responses to natural infection and immunity post vaccination. Moreover, computational modeling allows predicting plasma neutralization capacity from ABCORA seroprofiling results, obviating the need to conduct labor-intensive neutralization tests in this large-scale study.

The study will provide important data concerning the immune response after a SARS-CoV-2 vaccination in immunosuppressed patients, a patient population that was not, or only to a very limited extent, considered in the approval studies [15, 24].

## Trial status

The trial received provisional approval from the ethical committee Nordwest- and Zentraschweiz, Switzerland (2021-000593), on 01 April 2021 and final approval on 19 April 2021. The first patient was vaccinated in this study on 19 April 2021. This manuscript was submitted to the journal *Trials* on 17 May 2021; on this date, a total of 371 patients were randomized and received the first vaccine, and none of the patients received the second vaccine. We assume that the primary endpoint will be collected for all patients by end of August 2021.

## Data Availability

All local investigators have full access to the databases of the SHCS and STCS and provided with regular data updates by the cohort data centers. The trial platform infrastructure will be open to all investigators to be used for additional studies. Data from the first sub-study protocol will be available to local investigators following completion and publication of the main-results from the trial.

## Abbreviations

BOD: Burden of disease
CEC: Competent ethics committee
COVID: Corona virus diseases
CRF: Case report form
eCRF: Electronic case report form
EDTA blood: Ethylenediaminetetraacetic acid blood
FDA: Food and drug administration
HIV: Human immunodeficiency virus
IMV: Institute of medical virology
ITT: Intention to treat
PCR: Polymerase chain reaction
SARS-CoV-2: Severe acute respiratory syndrome coronavirus 2
SHCS: Swiss HIV Cohort Study
STCS: Swiss Tansplant Cohort Study
